# Using persistent homology as preprocessing of early warning signals for critical transition in flood

**DOI:** 10.1038/s41598-021-86739-5

**Published:** 2021-03-31

**Authors:** Syed Mohamad Sadiq Syed Musa, Mohd Salmi Md Noorani, Fatimah Abdul Razak, Munira Ismail, Mohd Almie Alias, Saiful Izzuan Hussain

**Affiliations:** Department of Mathematical Sciences, Faculty of Science and Technology, Universiti Kebangsaan Malaysia, 43600 Bangi, Selangor Malaysia

**Keywords:** Hydrology, Applied mathematics

## Abstract

Flood early warning systems (FLEWSs) contribute remarkably to reducing economic and life losses during a flood. The theory of critical slowing down (CSD) has been successfully used as a generic indicator of early warning signals in various fields. A new tool called persistent homology (PH) was recently introduced for data analysis. PH employs a qualitative approach to assess a data set and provide new information on the topological features of the data set. In the present paper, we propose the use of PH as a preprocessing step to achieve a FLEWS through CSD. We test our proposal on water level data of the Kelantan River, which tends to flood nearly every year. The results suggest that the new information obtained by PH exhibits CSD and, therefore, can be used as a signal for a FLEWS. Further analysis of the signal, we manage to establish an early warning signal for ten of the twelve flood events recorded in the river; the two other events are detected on the first day of the flood. Finally, we compare our results with those of a FLEWS constructed directly from water level data and find that FLEWS via PH creates fewer false alarms than the conventional technique.

## Introduction

Flooding is one of the most destructive natural disasters. Flooding occurs worldwide and often results in a high number of deaths and massive losses of property. Historical records show that the impact of flooding on human livelihood is unavoidable. However, while flood events are unavoidable, flood early warning systems (FLEWSs) may help predict when the next flood may occur. Hydrologists have long studied FLEWSs or attempted to forecast floods by constructing models of hydrological processes. An excellent review on worldwide FLEWS issues and techniques can be found in Ref.^1^ and Malaysia FLEWS in Ref.^2^.

Because flooding is generally defined as an overflowing of water onto land that is normally dry, an understanding of observational and historical water levels is important because such data provide climatic indicators for flooding. Much work has been done to construct an optimal method for water level forecasting that can alert authorities to the potential occurrence of a flood^3–10^; these efforts include statistical modeling, machine learning, fuzzy analysis, and extreme machine learning. These methods involve prediction of water level data to obtain warning signals.

Several fields of scientific research have suggested the existence of generic early warning signals as an indicator of systems approaching their critical tipping point^11^. These generic indicators are related to theory of critical slowing down (CSD)^12^. Theory of CSD states that the time series of indicator shows an increasing trend as a tipping point is approached^13,14^. Two possible CSD indicators of early warning signals include increases in variance^15^ and spectral density^16^. The significant aspect of CSD is that it captured hazard symptoms based on historical data. Therefore, early warning signal can be achieved without the need for prediction. This theory has been successfully used to capture the essence of shifts at tipping points in a wide range of systems ranging from ecosystems, to financial systems and the climate, detailed application can be found in refs.^17–24^. In our previous study, we showed that CSD theory is able to provide early warning signals of flood on the basis of the water level data of Kelantan River^25^. The need to develop a better FLEWS remains an urgent concern on account of the increasing intensity of floods.

Topological data analysis (TDA)^26,27^ provides a new approach to seek information from a data set based on a qualitative approach. TDA uses ideas and results from geometry and topology to study qualitative features or structures of data. Persistent homology (PH) is a new tool in TDA that can provide a precise description of qualitative features throughout their time evolution. PH is based on algebraic topology, which provides a well-understood theoretical framework with which to study the qualitative or topological features of data with complex structures. The key advantage of PH is that it is robust with respect to small perturbations in input data. Also, it is appealing for application for complex data set due to noise, high dimensionality or incomplete structure. These properties will be practical to deal with real-world data. A number of PH techniques have been applied to diverse problems, including spatial data clustering^28^, complex dynamical systems^29^, financial systems^23^, chemical and biological systems^30,31^. In particular, the exploration and application of TDA to the time delay embedding of a time series to model and classify dynamical systems and time-varying events has received great interest^28,32^.

Motivated by this new analytical tool and the existing need to develop better FLEWSs, in this paper, we propose the use of PH as a preprocessing step to obtain a FLEWS via CSD. We test our idea on the time series data of daily water levels collected at the Guillemard Bridge station, Kelantan River, Malaysia, from 01/01/2000 to 13/10/2010. We use PH to extract the topological features of the water level data and develop a new signal with flood information. Then, CSD indicators will be calculated on the PH signal to obtain flood early warning signals. The result suggests that the PH signal exhibit CSD by demonstrating increasing pattern near flood events in the CSD indicators. We then use quantile estimation to verify the increase pattern and obtain dates for flood signals. Quantile estimation is a method in extreme value theory that has been used by hydrologists to study rare events and extreme values^33,34^. Finally, we compare the results of FLEWS via PH with those of a FLEWS arising directly from the water level data.

In “Methods”, we provide a concise and informal review of the proposed early warning system (EWS) using CSD and the PH methodology for time series processing. In “Data” we introduce our water level data. “Results and discussions” presents our analysis and results, and “Conclusion” concludes the paper. All computations in this paper are conducted in the R-package TDA^35^.

## Methods

This section provides an introduction of EWS using CSD and basic concepts of PH processing for time series. Specifically, for PH processing pipeline we describe the reconstruction of the phase space and construct simplicial complexes and topological summaries of PH. Discussions on EWS using CSD and PH are provided by Scheffer et al*.*^11^ and Edelsbrunner and Harer^36^, respectively. Summary of the methodology implemented in this research is visualize as a flow chart in Appendix.

### Early warning system using critical slowing down

EWS is a tool consisting of a series of mechanisms and procedures used to detect hazards and monitor indicators, warning communications, and alarms. Previous researchers provided efficient EWSs for various disasters. However, developing an EWS based on real data is challenging and may lead to both false positive and false negative results. False negatives are situations in which a sudden shift occurs, but no early warning signal could be detected before the shift. False positives refer to situations in which a supposed early warning signal is not the result of an approaching critical point; this result is also called a false alarm. Therefore, improving the knowledge and applications of EWSs to obtain high-performance systems is necessary.

Scientific work in various fields has suggested that CSD is a valid indicator for EWS. Such slowing down, which is measured as increases in variance^15^ and spectral density^16^, may be shown to be a typical characteristic of a system approaching its tipping point. In the earth system, abrupt shifts in ocean circulation or climate may occur. Explanations for these abrupt climate changes usually invoke the existence of thresholds in external conditions where the climate system reaches its critical tipping point. A recent analysis revealed that the significant increase in each of eight ancient abrupt climate changes was preceded by a characteristic CSD of the fluctuation beginning well before the actual shift^13^.

Our previous study using water level data of Kelantan River suggested that CSD could produce an early warning signal for flood detection^25^ because ten of twelve flood events were preceded by an early warning signal while the two other events were detected on the first day of the flood. Note that, throughout this study, the term of an early warning signal is meant by any signal that is determined before the day of the flood event. By this definition, signals detected on the day of the event are not considered as an early warning signal but labeled as detection on the first day. However, the FLEWS produced also created six false alarms (signals that are detected during period with no recorded flood event). Therefore, in this paper, we add a preprocessing step to our pipeline method using PH to obtain a better FLEWS. We believe that the new information extracted from water level data by PH can be used as a basis for FLEWS. The next section will discuss basic concepts of PH for time series analysis.

### Reconstruction of the phase space

PH is a method that extracts the topological features of a data set that are not readily available in a time series in its standard form. Therefore, we use Takens’ embedding theorem^37^ to prepare the data set. Takens’ embedding theorem states that a time series can be used to reconstruct the phase space of the associated dynamical system and yield point cloud data. Given a time series $x_{1} ,x_{2} , \ldots ,x_{N}$, the constructed phase space consists of vectors

1 $$ x_{n} \left( {m,\tau } \right) = \left( {x_{n} ,x_{n + \tau } , \ldots , x_{{n + \left( {m - 1} \right)\tau }} } \right), $$

where $m$ is the embedding dimension and $\tau$ is the time delay. In this research, we set $\tau = 1$ and $m = 2$ because our experience indicates that these values give good analytical results. Other studies using these values have also obtained good results^25,29^. Thus, we obtain two-dimensional point cloud data from the reconstruction of the phase space. We can then use PH to extract two forms of topological features, namely, connected components (zero-dimensional topological features) and holes (one-dimensional topological features).

### Simplicial complexes

**Figure 1:**
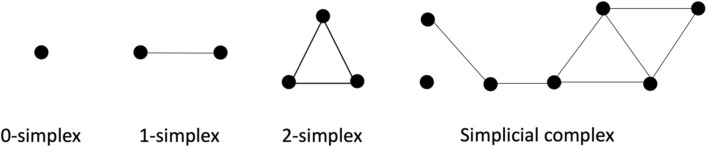
Construction of a simplicial complex using simplices.

The goal of PH is to analyze the topological features of a data set. Using the point cloud data obtained through the reconstruction of the phase space, we construct simplicial complexes. The building blocks for a simplicial complex are called k-simplexes, such as 0-simplex (vertex), 1-simplex (edge), and 2-simplex (triangle), as illustrated in Fig. 1.

**Figure 2:**
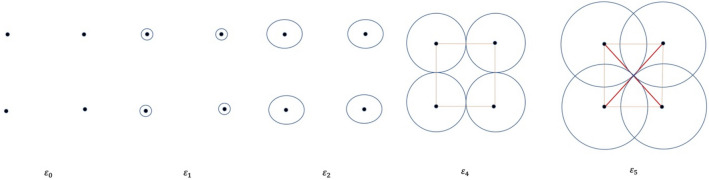
Filtered simplicial complex.

The idea of a filtered simplicial complex is used to analyze the evolution of topological features, particularly in terms of their appearance and disappearance. For example, consider the set of points in ${\mathbb{R}}^{2}$ shown in Fig. 2 and let $\varepsilon$ be a positive real number that is interpreted as a filtration parameter. By considering a $\varepsilon$ ball at each data point, we build an edge (1-simplex) between two points $a$ and $b$ if and only if the distance between them is less than $\varepsilon$. Similarly, we build a triangle (2-simplex) if and only if the pairwise distances between three points are each less than $\varepsilon$. These operations will produce the filtered simplicial complexes illustrated in Fig. 2. This type of simplicial complex is known as a filtered Vietoris–Rips simplicial complex or Rips complex^36^.

Rips complexes provides information of basic topological features, such as the number of components and holes. Algebraic topology captures these topological features by counting the rank of each homology group of the simplicial complex. For example, algebraic topology can compute the $k$-dimensional homology $H_{k} \left( X \right)$ for each natural number $k \in \left\{ {0,1,2, \ldots } \right\}$ of each simplicial Complex $X$. The rank of the zero-dimensional homology group $H_{0} \left( X \right)$ counts the number of connected components, the rank of the one-dimensional homology group $H_{1} \left( X \right)$ counts the number of holes and so on. These ranks of homology groups are also known as Betti numbers, i.e., the $p$th Betti number counts the number of $p$-dimensional holes. Because the simplicial complexes evolve when the value of the filtration parameter increases, PH detects which topological features persist across the scale.

Figure 2 shows an example of a filtered simplicial complex for point cloud data consisting of four points in a rectangle. At filtration parameter $\varepsilon_{0}$, four components exist, i.e., $H_{0} \left( X \right) = 4$. These components persist through filtration values $\varepsilon_{1}$ and $\varepsilon_{2}$. At filtration value $\varepsilon_{3}$, edges or 1-simplexes are formed; these simplexes join all of the points together into a single connected component and, hence, change the Betti number of the connected component to $H_{0} \left( X \right) = 1$. The connected component does not disappear as the filtration parameter is further increased. At filtration value $\varepsilon_{3}$, a one-dimensional hole in the data is born as the edges form a rectangle that gives $H_{1} \left( X \right) = 1$. The one-dimensional hole dies out at filtration parameter $\varepsilon_{4}$ when 2-simplexes or triangles appear.

### Topological summaries

Besides the construction of filtered simplicial complexes, PH provides us with information on the evolution of topological features existing in a data set. Several tools to summarize all of the information captured by PH are available. These topological summaries provide a concise description of the evolution of topological features across the scale. The precise information stored by these topological summaries includes the birth, growth, and death of all topological features.

The first topological summary is known as a persistence diagram. Persistence diagrams are a finite multiset of all birth–death pairs of topological features points in the extended $\overline{{{\mathbb{R}}^{2} }}$ plane, where ${\overline{\mathbb{R}}} = {\mathbb{R}} \cup \left\{ \infty \right\}$. The birth–death pair of a topological feature is written as point $\left( {\varepsilon_{i} ,\varepsilon_{j} } \right)$, where $\varepsilon_{i}$ refers to the birth point, $\varepsilon_{j}$ refers death point of the topological feature, and $j > i$. If the topological feature lives forever, we represent its birth–death point by the interval $\left( {\varepsilon_{i} ,\varepsilon_{\infty } } \right)$. A diagonal line in which all points on the line are born and die at the same time (i.e., each of the points on the diagonal has infinite multiplicity) exists in the persistence diagram. This diagonal line can help reveal which topological features are persistent. Points that lie close to the diagonal line indicate that the topological feature is not persistent, while points that lie far from the diagonal line correspond to persistent topological features.

Unfortunately, persistence diagrams are difficult to work with from the point of view of statistics and machine learning^38^. Therefore, we use another topological summary known as a persistence landscape^39^. Persistence landscapes are obtained by embedding the space of the persistence diagram into a function space. The advantage of a persistence landscape over a persistence diagram is that the former is a function, which means we can use the vector space structure of its underlying function space. Persistence landscapes also store all information obtained from persistence diagrams.

We now review the definition of persistence landscapes and the norm of a persistence landscape^38^. First, for a birth–death pair $\left( {b,d} \right)$, the piecewise linear function $f_{{\left( {b,d} \right)}} :{\mathbb{R}} \to \left[ {0,\infty } \right]$ is defined as follows:

2 $$ f_{{\left( {b,d} \right)}} = \left\{ {\begin{array}{*{20}c} {0, if\, x \notin \left( {b,d} \right)} \\ {x - b, if\, x \in \left. {\left( {b,\frac{b + d}{2}} \right.} \right)} \\ { - x + d,  if \, x \in \left( {\frac{b + d}{2},d} \right)} \\ \end{array} }\right., $$

The persistence landscape of the birth–death pairs $\left\{ {\left( {b_{i} ,d_{i} } \right)} \right\}_{i = 1}^{n}$ is the sequence of functions $\lambda_{k} :{\mathbb{R}} \to \left[ {0,\infty } \right], k = 1,2,3, \ldots$ where $\lambda_{k}$ is the $k$-th largest value of $\left\{ {f_{{\left( {b_{i} ,d_{i} } \right)}} \left( x \right)} \right\}_{i = 1}^{n}$. We set $\lambda_{k} \left( x \right) = 0$ if the $k$-th largest value does not exist; thus, $\lambda_{k} = 0$ for $k > n$. Similarly, the persistence landscape is a function $\lambda : {\mathbb{N}}{\text{x }}{\mathbb{R}} \to \left[ {0,\infty } \right]$, where $\lambda \left( {k,t} \right) = \lambda_{k} \left( t \right)$. In this definition, the assumption that $b$ and $d$ are finite is considered. For cases where $b$ and/or $d$ are infinite, see Bubenik and Dłotko^38^.

Given a set of persistence landscapes, $\lambda^{\left( 1 \right)} , \ldots , \lambda^{\left( N \right)}$, the average persistence landscape, $\overline{\lambda }$, is defined pointwise, $\overline{\lambda } = \frac{1}{N}\sum\nolimits_{i = 1}^{N} {\lambda_{k}^{\left( i \right)} \left( t \right)}$. The distance between persistence landscapes and between average persistence landscapes can be given by using the $L^{\infty }$ norm,

3 $$\left|\left| \lambda - \lambda^{\prime}\right|\right|_{\infty }= \begin{array}{*{20}c} {sup} \\ {k,t} \\ \end{array} \left| {\lambda_{k} \left( t \right) - \lambda_{k}^{^{\prime}} \left( t \right)} \right|, $$

or the $L^{p}$ norm; for $1 \le p < \infty$,

4 $$\left|\left| \lambda - \lambda^{\prime}\right|\right|_{p} = \left[ {\mathop \sum \limits_{k = 1}^{\infty } \smallint \left| {\lambda_{k} \left( t \right) - \lambda_{k}^{^{\prime}} \left( t \right)} \right|^{p} dt} \right]^{\frac{1}{p}} , $$

In Bubenik^39^, the persistence landscape is stable with respect to the $L^{p}$ distance when $1 \le p < \infty$. That is, under the hypothesis, sufficiently small perturbations of a function under the supremum norm led to small changes in the persistence landscape of the PH of the sublevel sets of that function under the $L^{p}$ norm. In the upcoming computation, we will only consider the $L^{1}$ norm.

**Figure 3:**
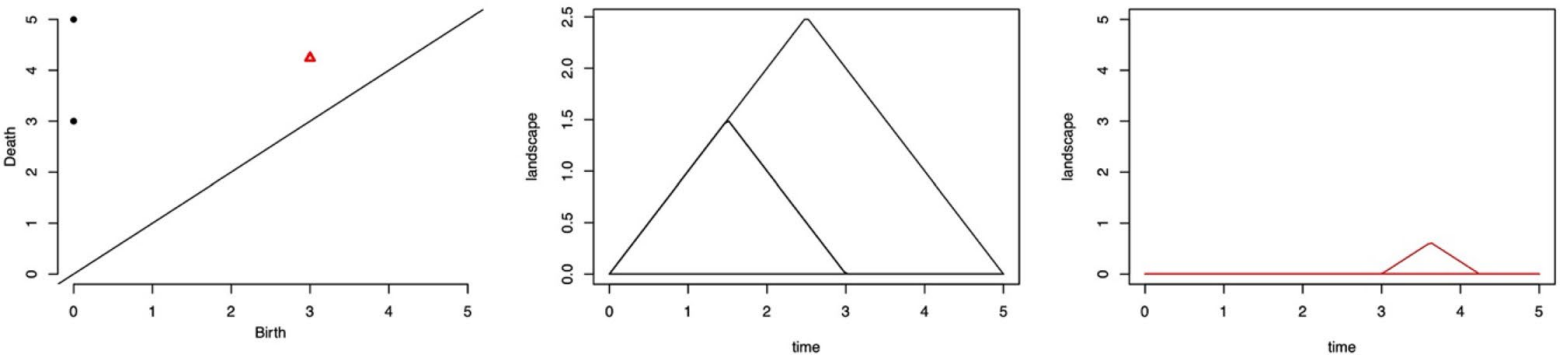
Topological summaries of the point-cloud data in Fig. 2—persistence diagram (left) and persistence landscape for connected components (middle) and holes (right).

Figure 3 shows the topological summaries for the point cloud data in Fig. 2. The left-most column shows a persistence diagram. The black dots on the persistence diagram correspond to connected components (zero-dimensional topological features), and the red triangle corresponds to a hole (one-dimensional topological feature). The middle and right-most topological summaries reveal the persistence landscapes obtained from the persistence diagram. The middle persistence landscape represents connected components, and the right-most persistence landscape represents a hole.

## Data

The climate in Malaysia is governed by two main regimes, namely, the southwest and northeast monsoons^40^. The southwest monsoon takes place between May to August and is responsible for the dry period of the whole country. The northeast monsoon usually begins in November, ends in February, and is responsible for the wet period in the eastern coast of Peninsular Malaysia. This wet period is often manifested as heavy rains, which frequently cause monsoon flooding.

Kelantan, a state located at the eastern coast of Peninsular Malaysia, is often affected by monsoon flooding. Kelantan River is one of the main rivers of the state; it is situated in the northeastern portion of Peninsular Malaysia between the latitudes $4^{^\circ } 40^{^{\prime}}$ and $6^{^\circ } 12^{^{\prime}}$ N and longitudes $101^{^\circ } 20^{^{\prime}}$ and $102^{^\circ } 20^{^{\prime}}$ E. At 248 km long, it is the longest river in Kelantan and drains an area of 13,100 ${\text{km}}^{2}$. The total area of Kelantan is 15,022 ${\text{km}}^{2}$, and approximately 68.5% of the population in the area lives in the Kelantan River Basin. The basin has an annual precipitation about 0 mm in the dry season from March to May and 1750 mm in the rainy or monsoon season from November to January. The estimated runoff of the Kelantan River measured at the Guillemard Bridge is 557.5 m^3^ s^−1^.

The Kelantan River originates from the Ulu Sepat mountain, moving northwards passing through such major towns as Kuala Krai, Tanah Merah, Pasir Mas and Kota Bharu, before finally discharging into the South China Sea. It divides into the Galas River and Lebir River near Kuala Krai, about 100 km from the river mouth. The Galas River has two main tributaries (the Nenggiri River and the Pergau River), while the Lebir River has one major tributary (the Relai River). About 95% of the catchment is steep mountainous country rising to a height of 2135 m which dominated by sedentary soils. While on riverine floodplains and low riverine terraces, alluvial soil appears. For the land use, the mountainous areas are covered with virgin jungle while agriculture (rubber, paddy and palm oil) are planted in the lowlands. A recent study on Kelantan River basin regarding land use and climate change^41^ (i.e., precipitation) reported that both of the variables are factor for floods, however precipitation changes are the main driver.

Based on the report by the Department of Irrigation and Drainage (DID), Malaysia, on the flood at Kelantan^42^, starting with the year 2000, the first severe flood that hit Kelantan was reported in December 2001 due to unusual tropical cyclone Vamei. Afterwards, in the year 2007 and 2009, heavy rainfall again had triggered major floods in Kelantan. To date, the worst flood reported in Kelantan was at the end of 2014, commonly known as the Kelantan Big Yellow Flood 2014^43^. DID has assigned sixteen meters as the danger water level of Kelantan River at Guillemard Bridge station; water levels reaching this height are a good indicator of potential flooding.

**Figure 4:**
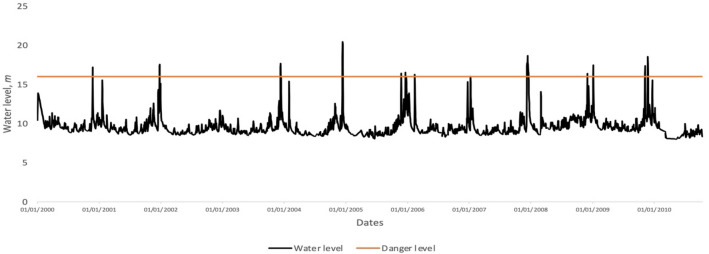
Time series plot of the daily water level data of Kelantan River obtained at Guillemard Bridge station from 01/01/2000 to 13/10/2010.

In this research, our analysis focuses on water levels in Kelantan River. The daily water level data of Kelantan River recorded at the Guillemard Bridge station (measured in meters, $m$) were obtained from the DID. Figure 4 shows a time series plot of the daily water level data of Kelantan River obtained from 01/01/2000 to 13/10/2010. Some important statistical parameters of the time series are shown in Table 1. Table 2 lists the dates in which the water level data exceeded the danger level (16 m); these dates will be used as benchmark dates for flood events.

**Table 1:** Statistics of the time series of the daily water level data of Kelantan River obtained at Guillemard Bridge station from 01/01/2000 to 13/10/2010.

Statistics	Daily
Number of data	3939
Average	9.52
Max	20.44
Min	8
Standard deviation	1.26
Skew	3.29
Kurtosis	15.64

**Table 2:** Dates in which the water level data exceeded the danger water level (16 m) of Kelantan River—the data are obtained from the Guillemard Bridge station from 01/01/2000 to 13/10/2010.

No	Date of flood events	No	Date of flood events
1	23/11/2000	7	12/02/2006–13/02/2006
2	24/12/2001–25/12/2001	8	08/01/2007
3	10/12/2003–11/12/2003	9	08/12/2007–18/12/2007
4	11/12/2004–14/12/2004	10	30/11/2008
5	24/11/2005	11	04/01/2009–05/01/2009
6	18/12/2005	12	06/11/2009–07/11/2009

## Results and discussions

Our results and discussion are divided into three parts. The first part describes the results obtained by applying PH to the water level data of Kelantan River. This part of the results investigates the evolution of topological features and quantifies the changes observed mathematically. In the second part of the results, we use the signal obtained from PH to search for a flood early warning signal using the CSD theory. Here, we found that PH signal exhibits CSD by demonstrating an increasing pattern near all flood events in the CSD indicators. Therefore, the new signal can be use as a basis for FLEWS. Then, in the last part of this section, we use quantile estimation to study potential CSD indicators and develop a FLEWS. A comparison of the results obtained through EWS with PH preprocessing and EWS arising directly from the water level data is also conducted.

### Persistent homology of water level data

This section describes the results of our reconstruction of the phase space, persistence diagrams, and persistence landscapes obtained by applying PH to the daily water level data of Kelantan River. Using the time series of water level data, we perform Takens’ time-delay embedding with $\tau = 1$ and $m = 2$. We then obtain two-dimensional point cloud data from the reconstruction of the phase space. Because our objective is to analyze the data based on the daily evolution of topological features, we introduce the concept of sliding windows. The size of the sliding windows used in this work has a length of ten days with $\tau = 1$. Hence, the input data for PH take the form of windows of the two-dimensional point cloud data with a length of ten. Each window is denoted by the end date of the data included in the window; thus, the output for each window is obtained from the data of previous dates only. PH is performed on this time-ordered sequence of sliding windows to study the daily evolution of topological features.

**Figure 5:**
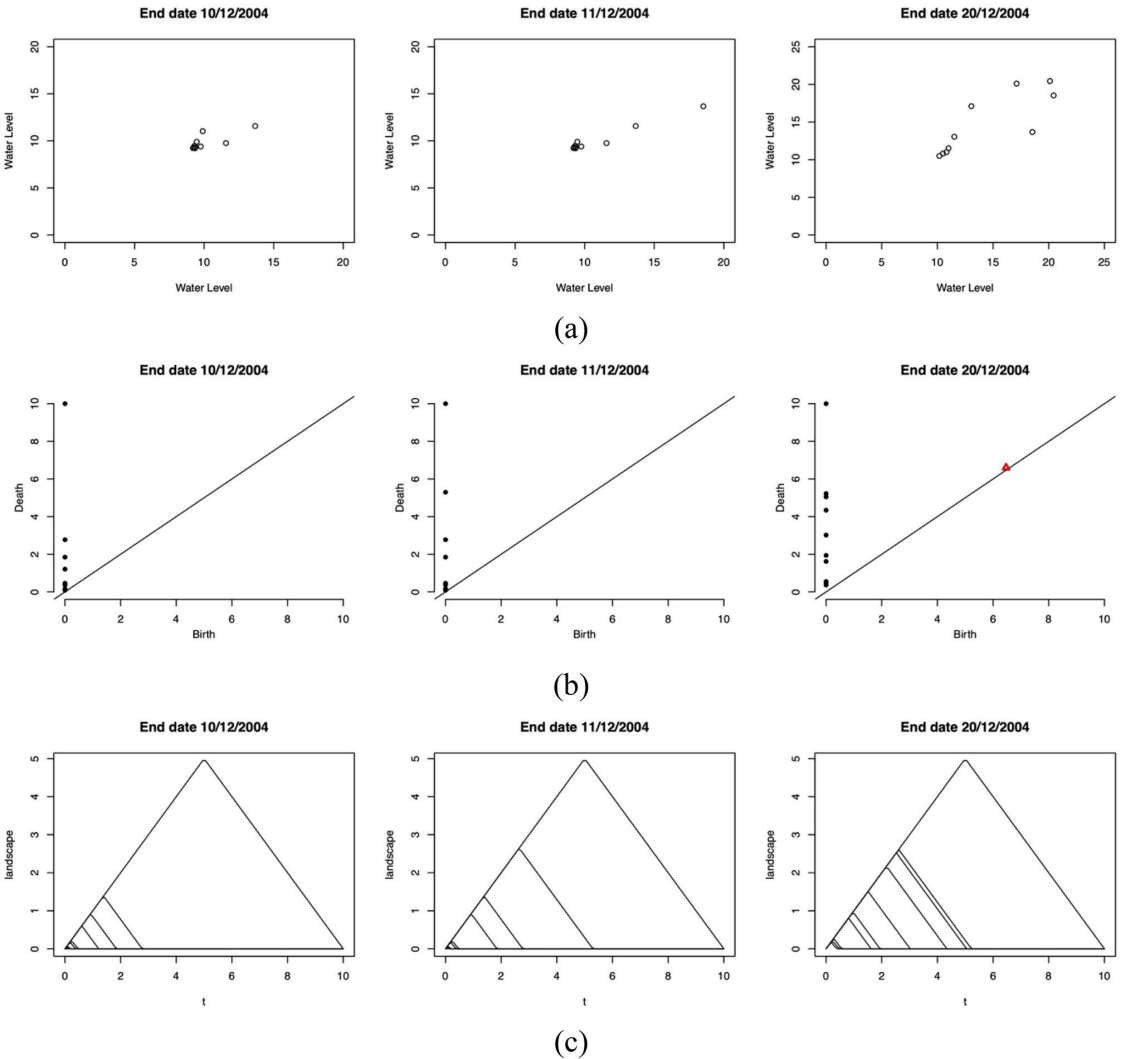
(**a**) Reconstructed phase space, (**b**) persistence diagrams, and (**c**) zero-dimensional persistence landscapes for windows with selected end dates during the flood events of 2004.

We visualize the results from PH by showing the evolution of topological features for the windows of one day before a flood, the first day of the flood, and the tenth day of the flood. Here we illustrate our results for the flood event of December 2004 because the highest water level reading in our data set (20.44 m) was obtained during this flood event (12/12/2004; Fig. 4). The evolution of the reconstructed phase space, persistence diagrams, and persistence landscapes of the flood event in December 2004 is shown in Fig. 5. The top row of Fig. 5 shows the reconstructed phase space of three different windows with end dates 10/12/2004 (one day before flood), 11/12/2004 (first day of the flood), and 20/12/2004 (tenth day of the flood). In the window with end date 10/12/2004, the points in the reconstructed phase space are densely packed together, which indicates that the water level in this window are in the same range. The reconstructed phase space for the window with end date 11/12/2004 is very similar to that of the window with end date 10/12/2004, except for the existence of one point located slightly far from the densely packed points. This point indicates the beginning of the development of extreme values of the water level data. The window with end date 20/12/2004 shows scattered points, which indicates the presence of high values of water level data with a wide range.

The corresponding persistence diagrams for each reconstructed phase space of the windows is shown in the middle row of Fig. 5. The persistence diagram for the window with end date 10/12/2004 shows only black dots located near the origin and diagonal line, which indicates the presence of short-lived (non-persistent) connected components. The persistence diagram for the window with end date 11/12/2004 also consists of only black dots, but one dot has a y-coordinate that is higher than those of the other dots; this finding indicates the existence of one long-lived (persistent) connected component. For the window with end date 20/12/2004, more black dots located far from the origin and diagonal line are observed; these dots indicate the presence of a larger number of long-lived connected components in this window. In the window with end date 20/12/2004, a red triangle is located on the diagonal line; this triangle reflects the existence of a short-lived hole in the point cloud data of the window. Note that for all three windows, a black dot is observed at the highest value of the x-coordinate (i.e., the maximum filtration value). This black dot reveals that, once the graph is fully connected into a single connected component, it remains fully connected (i.e., the component never dies) as the filtration value is further increased.

We only visualize the zero-dimensional persistence landscape because we find that the existence of one-dimensional topological features (i.e., holes) does not remarkably affect the results as most of the windows do not contain this feature. The bottom row of Fig. 5 illustrates the zero-dimensional persistence landscapes for the corresponding reconstructed phase space and persistence diagrams. The zero-dimensional persistence landscape for the window with end date 10/12/2004 consists of only a small sequence of functions (bottom left). The zero-dimensional persistence landscape for the window with end date 11/12/2004 has one landscape function with an intermediate value (bottom middle). The persistence landscape for the window with end date 20/12/2004 has a zero-dimensional persistence landscape that is more complex than those for the two other windows and shows greater landscape functions. This evolution of the zero-dimensional persistence landscapes can be further quantified by calculating the norm of the persistence landscapes.

**Figure 6:**
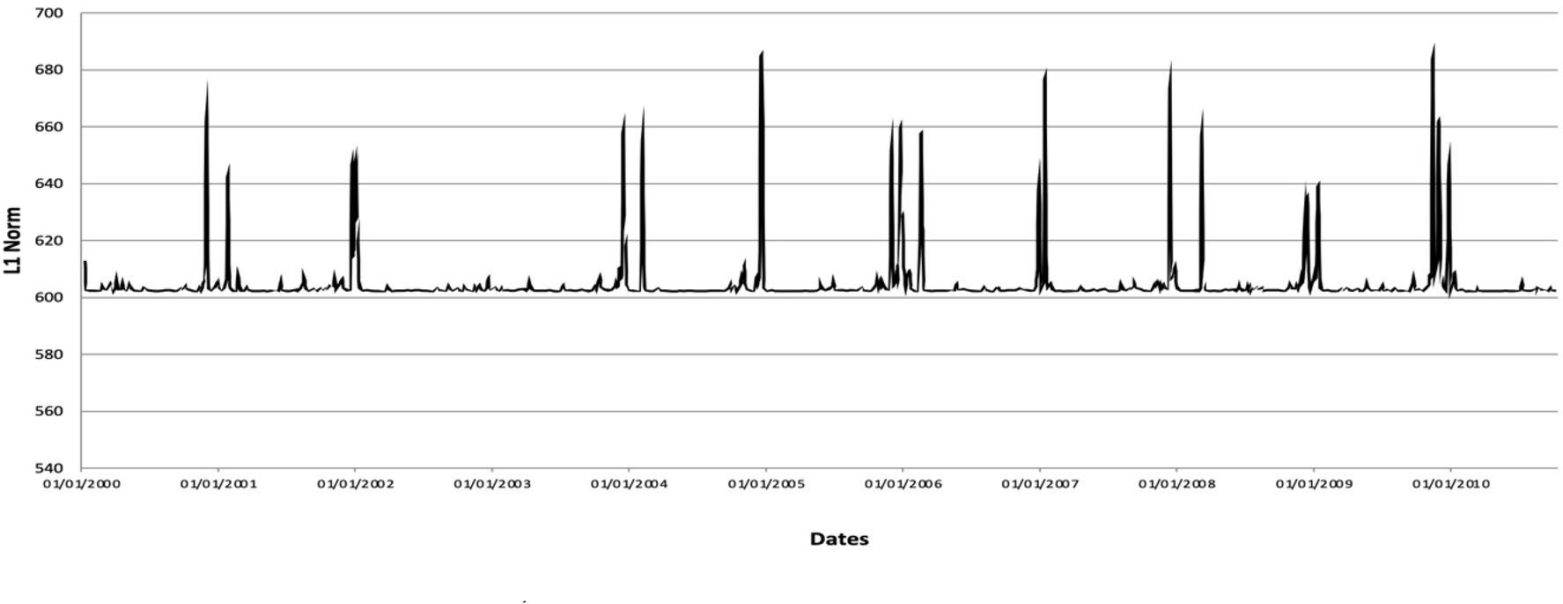
Time series of the *L*^1^ norm of the zero-dimensional persistence landscapes of the time-ordered sequence of sliding windows.

Figure 6 shows the time series of the $L^{1}$ norm of the zero-dimensional persistence landscapes of the time-ordered sequence of sliding windows of the water level data of Kelantan River obtained at Guillemard Bridge station from 11/01/2000 to 13/10/2010. The time series of the $L^{1}$ norm illustrates the daily evolution of topological features. The time series of the $L^{1}$ norm of the zero-dimensional persistence landscapes is clearly consistent with the original time series of the water level data (Fig. 4). Peaks in the time series of the $L^{1}$ norm may also be observed near the recorded flood events, which suggest that this time series contains information regarding the flood events of Kelantan River. Therefore, we use the time series of the $L^{1}$ norm of the zero-dimensional persistence landscapes as a signal to search for an early warning signal of flood events at Kelantan River.

### Persistent homology signal exhibiting critical slowing down

Now that the time series of the $L^{1}$ norm of the zero-dimensional persistence landscapes has been established, we apply the generic early warning signal indicators of CSD to test for an early warning signal. To this end, we quantify temporal variations in the persistence of connected components in the time-ordered set of the zero-dimensional persistence landscapes. We employ sliding windows with a length of ten days and a sliding step of one day to calculate the variance and average spectral density at low frequencies of the $L^{1}$ norm time series. These windows should not be confused with the sliding windows of length ten days used earlier to reconstruct the phase space throughout the computation of the time series of the $L^{1}$ norm of the persistence landscapes.

**Figure 7:**
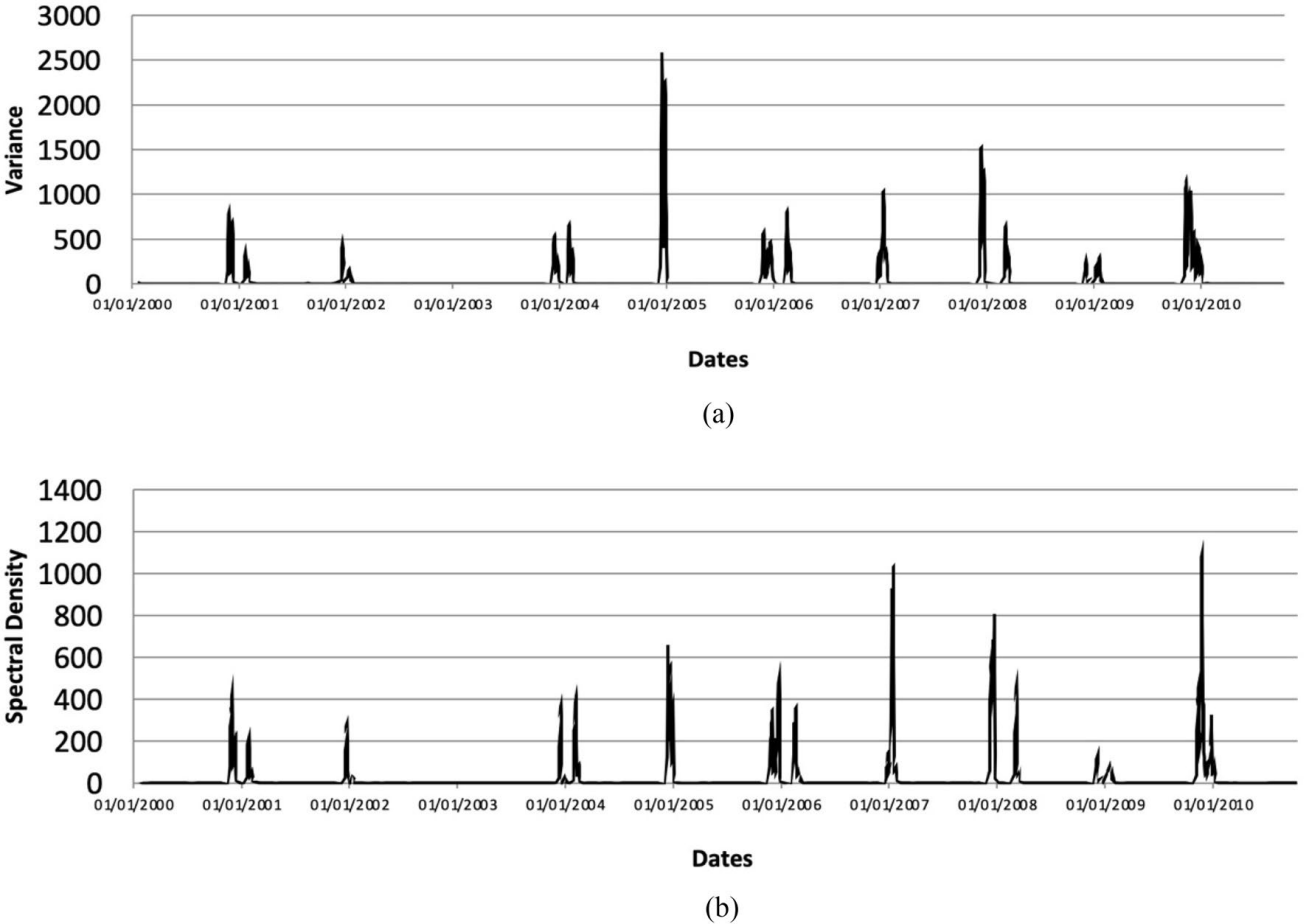
Time series of CSD indicators for early warning signals based on the PH framework—(**a**) variance and (**b**) average spectral density at low frequencies of the time series of the $L^{1}$ norm of the zero-dimensional persistence landscapes.

**Figure 8:**
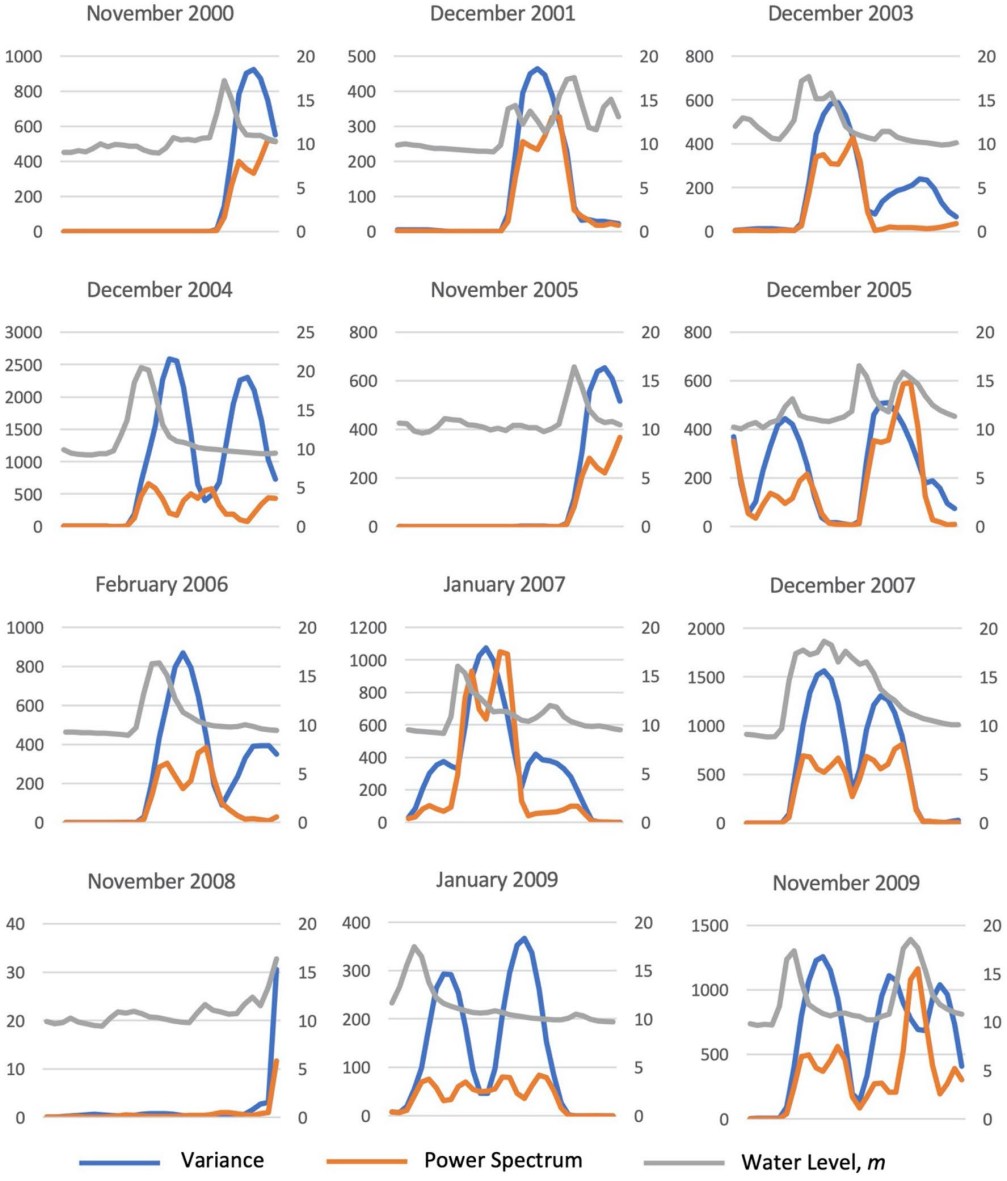
Trends of the time series of the CSD indicators (primary axis) variance and average spectral density at low frequencies and their respective water level time series (secondary axis) for all twelve flood events at Kelantan River from 01/01/2000 to 13/10/2010.

Figure 7 shows the obtained time series of the CSD indicators variance and average spectral density at low frequencies for EWS from the time series of the $L^{1}$ norm of the persistence landscapes. The time series of variance and average spectral density at low frequencies substantially increase around the flood events listed in Table 2. This finding verifies that the signal obtained from PH, i.e., the time series of the $L^{1}$ norm of the persistence landscapes, exhibits CSD. Figure 8 shows magnified plots of the CSD indicators (primary axis) for all twelve flood events at Kelantan River listed in Table 2 and their respective water level data (secondary axis). Both indicators show an increasing or fluctuation pattern near all of the flood events. Moreover, the increasing patterns of both indicators occur simultaneously or nearly simultaneously. These increasing patterns in the time series of the CSD indicators may be further examined to obtain signal for FLEWS.

### Flood early warning systems

To produce an EWS, we use quantile estimation. As the time series of the $L^{1}$ norm of the zero-dimensional persistence landscapes exhibits CSD, i.e., an increasing pattern near all flood events, we use quantile estimation to determine dates with a significantly increasing pattern of the identified CSD indicators. These dates will specify whether the signal obtained from the CSD indicators is an early warning signal, a late signal (flood detection only), or a false alarm. This procedure will create a FLEWS for Kelantan River.

The extraction of dates using quantile estimation will provide us with thresholds for the CSD indicators. When we peak over these thresholds, we will obtain dates with extreme values that are responsible for the increasing pattern of the indicators. Because our time series water level data may be considered short and only twelve flood events are included in the data set, we can list all possible values of quantiles and find the optimum quantile required to obtain the FLEWS. Table 3 lists the results of FLEWS from the time series of the $L^{1}$ norm of the persistence landscapes for all possible quantile values with their respective weights.

**Table 3:** List of possible quantile values for the FLEWS from the time series of the $L^{1}$ norm of the zero-dimensional persistence landscapes with their respective results and weights.

Events/quantile	10%	11%	*12%*	13%	14%	15%	16%	17%	18%	19%	20%
Early signal	5	8	*10*	10	10	10	11	12	12	12	12
Late signal	7	4	*2*	2	2	2	1	0	0	0	0
False alarm	3	3	*4*	4	6	10	10	12	14	16	17
Weight	4.0	4.6	*4.8*	4.8	4.4	3.6	3.8	3.6	3.2	2.8	2.6

We explored many numbers for these quantiles, but it seems that at the range 10% to 20% is where the optimum quantile may be found. Specifically, Table 3 shows that at 10% quantile, the number of the early signal obtained is five for the twelve actual flood events and it shows a non-effective FLEWS. So, there is no need to lower the number of the quantile value as it will only come out with a less effective FLEWS. At the quantile 20%, the number of false alarms is seventeen, which is more than total number of the actual flood events (twelve flood events). This number of false alarms also shows a non-efficient FLEWS. If we continue to increase the quantile value, it will only create a FLEWS with more false alarms. So, we can conclude that the optimum quantile is in the range 10% to 20% of the quantile values.

The optimum quantiles for the FLEWS from the time series of the $L^{1}$ norm of the zero-dimensional persistence landscapes is at 12% and 13%. This is because, at these quantiles, the outcome for early and late signal start to be stationary while the number of false alarms continue to raise. Further quantification by assigning weights for each outcome of the EWS results (i.e., 0.5 for early signal, 0.3 for late signal, and − 0.2 for false alarm) and calculation of the total weights of each quantile, the results justify our observation. The largest weight, which is 4.8, occurs at quantiles 12% and 13%. We thus choose the 12% quantile as the threshold because it is the quantile with the lowest value that produces the same results. Regarding the weight applied, the biggest weight of 0.5 is assigned to the outcome of early warning signals as it shows the effectiveness of the FLEWS. For a late signal (or detection), we assigned the weight 0.3 as this outcome is expected for some flood events. Lastly, we take the weight − 0.2 for false alarms, as these false alarms have a negative impact in terms of the efficiency of the EWS.

Table 4 shows the results of FLEWS from the $L^{1}$ norm of the zero-dimensional persistence landscapes at the optimum quantile of 12%; the results of FLEWS arising directly from the water level data at the optimum quantile 15% reported in Syed Musa et al.^25^, are also provided for comparison. The FLEWS from the $L^{1}$ norm of the zero-dimensional persistence landscapes at the optimum quantile with threshold values of 4.8991 for the time series of variance and 2.8203 for the time series of the average spectral density at low frequencies succeeds in producing ten early warning signals of the twelve actual flood events. The signals for the two other flood events are detected on the first day of the flood without an early signal.

**Table 4:** Results of FLEWS from the time series of the $L^{1}$ norm of the zero-dimensional persistence landscapes (with PH preprocessing) and FLEWS arising directly from the water level data with optimum thresholds of 12% and 15%, respectively.

Actual flood events	FLEWS via PH (12%)	FLEWS via water level (15%)
23/11/2000	22/11/2000 (early 1 day)	22/11/2000 (early 1 day)
None	19/01/2001 (false alarm)	19/01/2001 (false alarm)
None	No signal	16/11/2001 (false alarm)
24–25/12/2001	16/12/2001 (early 8 days)	16/12/2001 (early 8 days)
None	No signal	17/12/2002 (false alarm)
10–11/12/2003	02/12/2003 (early 8 days)	30/11/2003 (early 10 days)
None	29/01/2004 (false alarm)	30/01/2004 (false alarm)
None	29/10/2004 (false alarm)	No signal
11–14/12/2004	10/12/2004 (early 1 day)	10/12/2004 (early 1 day)
24/11/2005	23/11/2005 (early 1 day)	23/11/2005 (early 1 day)
18/12/2005	15/12/2005 (early 3 days)	15/12/2005 (early 3 days)
12–13/02/2006	11/02/2006 (early 1 day)	12/02/2006 (first day)
08/01/2007	22/12/2006 (early 17 days)	21/12/2006 (early 18 days)
None	No signal	04/11/2007 (false alarm)
08–18/12/2007	07/12/2007 (early 1 day)	07/12/2007 (early 1 day)
None	29/02/2008 (false alarm)	29/02/2008 (false alarm)
30/11/2008	30/11/2008 (first day)	29/11/2008 (early 1 day)
04–05/01/2009	28/12/2008 (early 7 days)	02/01/2009 (early 2 day)

The same results are obtained through the FLEWS arising directly from the time series of water level in Syed Musa et al.^38^. The optimum quantile for the FLEWS from the time series water level data is 15% with threshold values of 0.4742 and 0.7909 for the time series of variance and average spectral density at low frequencies, respectively. The advantage of applying PH and obtaining the time series of the $L^{1}$ norm of the zero-dimensional persistence landscapes in this research is that the FLEWS with PH preprocessing creates fewer false alarms; for example, the proposed system results in four false alarms, with rates of 25% compared with the six false alarms, with rates 33.33% created by the FLEWS arising directly from the water level data.

In details, in terms of early warning signals established, difference between FLEWS via PH and FLEWS via water level is just a couple of days’ ranges. For example, for flood event December 2003 FLEWS via water level provide an early warning two days before the FLEWS via PH, flood event January 2001 also FLEWS via water level provide an early warning one day ahead while for flood events January 2009, FLEWS via PH provide an early warning five days ahead of the FLEWS via water level. Another crucial results here when comparing both FLEWSs, we can see that there is a flood event in each FLEWS that gained better outcome compare to the other which is from detection on the first day of flood to early warning of one day ahead, which is flood event February 2006 for FLEWS via PH and flood event November 2008 for FLEWS via water level.

In terms of false alarms, there are three false alarms that both FLEWSs detected at the same time, false alarms January 2001, January 2004, and February 2008. This consistency on detection of those false alarms by both FLEWSs could bring more insights if further study. The extra false alarm that is detected solely by FLEWS via PH is in October 2004 where there is no flood event recorded during that period. While FLEWS via water level created two more extra false alarms in November 2001 and December 2002. Notice, results from these both FLEWSs could offer an enhanced FLEWS if they can be merge or applied together.

## Conclusion

This study investigates the application of PH and CSD theory to produce a reliable FLEWS. The proposed approach was tested on daily water level data of Kelantan River. PH was used to extract the signal of topological features from the water level date, and then CSD theory was applied to determine indicators for early warning signals. Quantile estimation was subsequently carried out to extract dates for flood signal. The following conclusions may be drawn from the results of this study:

1. PH could be successfully applied to the hydrological field as a preprocessing step to analyze water level data and provide early warnings of flood disasters at Kelantan River.
2. The signal of topological features obtained through PH exhibits CSD by demonstrating an increasing pattern of the time series indicators of CSD (i.e., variance and average power spectral density at low frequencies).
3. Quantile estimation is carried out on the basis of the increasing pattern of CSD indicators to establish early warning signals for ten out of twelve actual flood events at Kelantan River; the two other signals are detected on the first day of the actual flood event.
4. Using PH as a preprocessing step for FLEWS provides an advantage over FLEWS arising directly from the water level data by producing fewer false alarms.

In summary, this study provides a new framework that integrates PH and CSD to achieve a reliable FLEWS. This framework may lead to more extensive studies on FLEWSs, wider applications of PH, and better estimations of flood risk. The first drawback of this study, which can be strengthened for future studies is to consider other climatic parameter to the FLEWS to achieve more comprehensive model. Besides, if predicted water level with flood event data can be attained, it can be used as a validation set for the model. This will authenticate the full performance of the proposed methods.
